# Case Report: Cytomegalovirus-specific T-lymphocyte infusion for resistant cytomegalovirus retinitis

**DOI:** 10.3389/fopht.2023.1131674

**Published:** 2023-07-03

**Authors:** Tal Saban, Liat Shargian, Maya Eiger-Moscovitch, Susan Prockop, Ekatarina Doubrovina, Moshe Yeshurun, Michal Kramer

**Affiliations:** ^1^ Department of Ophthalmology, Rabin Medical Center - Beilinson Hospital, Petach Tikva, Israel; ^2^ Institute of Hematology, Davidoff Cancer Center, Rabin Medical Center - Beilinson Hospital, Petach Tikva, Israel; ^3^ Hadassah Faculty of Medicine, Hadassah University Hospital, The Hebrew University of Jerusalem, Jerusalem, Israel; ^4^ Department of Pediatrics, Memorial Sloan Kettering Cancer Center, New York, NY, United States; ^5^ Center for Immune Cellular Therapy, Memorial Sloan Kettering Cancer Center, New York, NY, United States; ^6^ Faculty of Medicine, Tel Aviv University, Tel Aviv, Israel

**Keywords:** CMV retinitis, T-lymphocytes infusion, resistant CMV retinitis, cytomegalovirus-specific T-lymphocyte, CMV-specific cytotoxic T lymphocytes

## Abstract

**Purpose:**

We hereby describe a case of persistent cytomegalovirus (CMV) viremia and retinitis following allogeneic hematopoietic cell transplantation (HCT) that was successfully treated with infusion of CMV-specific cytotoxic T lymphocytes (CTL) despite previous treatment with Epstein Bar Virus (EBV) -specific CTL, which occurred 5 months earlier.

**Observations:**

Following several anti- viral medication treatment trials that failed to eradicate the infectious process, the patient was treated with infusions of CMV-CTL from a biobank of cryopreserved virus-specific cells. Shortly after the first infusion, a remarkable response was noted. A few days after the second infusion, the retinitis resolved completely. No recurrence was noted at the one-year follow-up, and there was no evidence of GVHD.

**Conclusions and importance:**

The case is unique for two reasons: use of virus-specific CTL for an indication of CMV retinitis; and successive administration, in the same patient, of third-party virus-specific CTL to treat two different infections (Epstein-Barr virus and cytomegalovirus) on two separate occasions following hematopoietic cell transplantation.

## 1 Introduction

CMV infection following allogeneic HCT can cause major morbidity and mortality. Clinical manifestations include pneumonia, hepatitis, gastroenteritis, retinitis, and encephalitis (1–3). Anti-viral treatment is initiated pre-emptively when the CMV replication level rises above a defined threshold, and therapeutically, when end-organ disease is diagnosed. However, several considerations limit the use of these drugs, most notably their toxicity profile and the risk of emergence mutations leading to drug resistance (4).

T lymphocytes play an important role in anti-viral immunity. The use of cellular immune therapy based on virus-specific CTL in patients after allogeneic HCT has been investigated for more than two decades. The aim of this report was to describe a young man after allogeneic HCT who was treated with virus-specific T-lymphocytes for persistent CMV retinitis.

This case is unique since the patient was previously treated with CTL infusion directed against EBV. This is the first description of a single patient receiving CTL from two unrelated donors, directed for two different viruses for two separate clinical events.

## 2 Case description

### 2.1 Patient history

A 26-year-old male presented to the uveitis clinic of a tertiary medical center with left eye visual disturbance. One year previously, he had undergone allogeneic HCT to treat severe aplastic anemia.

Prior to HCT, both the donor and the recipient were tested for the presence of antibodies for EBV and CMV. The donor had negative serologic testing for both viruses, meaning he had never been exposed to these viruses and therefore never developed immunization memory against them. The recipient had positive serologic tests to both CMV and EBV.

The post-transplantation course was complicated by acute GVHD requiring prolonged immunosuppressive therapy. The resulting profound immunosuppressive state led to reactivation of EBV and subsequent development of diffuse large B-cell lymphoma. The immunosuppression was stopped, and treatment with chemo-immunotherapy was initiated, but the disease progressed. In an attempt to accelerate reconstitution of EBV-specific immunity, the patient was treated with third-party EBV CTL, with a sustained complete response. The EBV viral load was very high at the time of diagnosis of post-transplant lympho-proliferative disorder. By the time the patient was treated with rituximab and CHOP, the viral load was negative. As for CMV, the patient had persistent CMV viremia during the year before the current admission, with recent complaints of decreased vision in the left eye, despite continuous anti-viral treatment. Peripheral viral load of CMV, just before the last CMV-CTL was minimal, ranging from zero and 10,000 copies. Nevertheless, retinal disease was refractory to treatment.

### 2.2 Findings on admission

**Figure 1:**
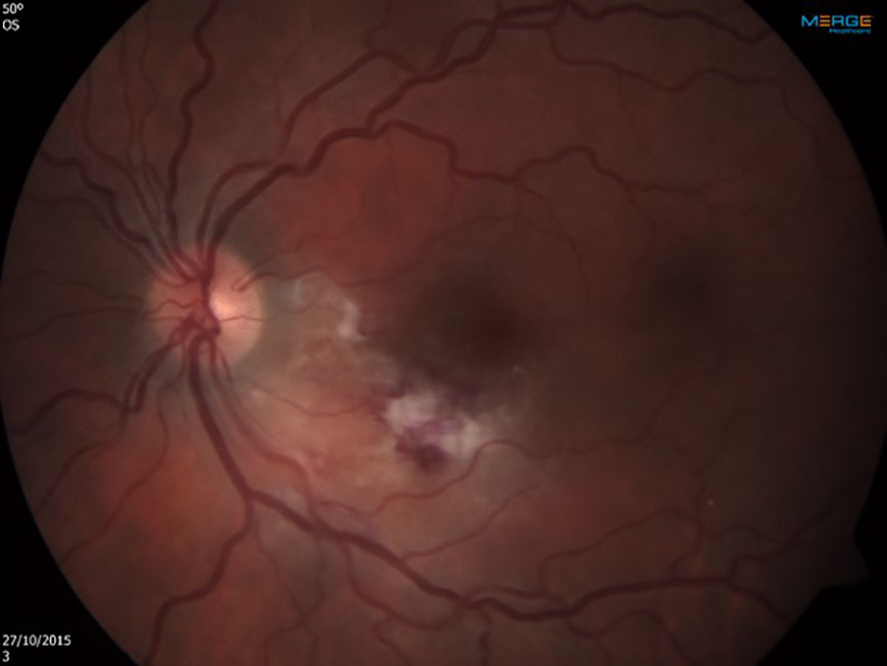
Left eye at presentation. Retinitis foci and several retinal exudates accompanied by flame-shaped retinal hemorrhages located inferior and nasal to the macula.

Upon admission a complete ophthalmologic examination was performed: Best corrected visual acuity was 6/6 in the right eye and 6/7 (5). in the left eye. There were no pathologic findings in the right eye. Exam of the left eye revealed retinitis foci adjacent to several retinal exudates and flame-shaped retinal haemorrhages located inferior and nasal to the macula (**Figure 1**). The retinal findings were imaged using OCT in order to locate the depth of the inflammatory process, the involved layers and the exact dimensions. A color fundus photography was used for further comparison. The option of performing an invasive ocular procedure for a PCR confirmation of the pathogen was considered. However, after assessing risks and benefits, in light of a classic ocular presentation of CMV retinitis alongside confirmed CMV viremia, a presumed diagnosis of CMV retinitis was made based on the clinical findings.

## 3 Management and clinical course

When retinitis was diagnosed in the presence of CMV viremia, CMV retinitis was the working diagnosis. The patient was treated with twice weekly intravitreal ganciclovir injections. Once the retinal lesions showed clinical regression, the intravitreal injections were tapered to once weekly. A sudden deterioration in the retinal state prompted the referral of the patient for an anterior chamber tap for polymerase chain reaction test. The results confirmed the presence of a new-onset UL-97 mutation which is associated with viral resistance to ganciclovir and valganciclovir. Accordingly, treatment was switched to twice weekly intravitreal injections of foscarnet. This led to a slow improvement of the retinitis, however, total resolution was not achieved.

**Figure 2:**
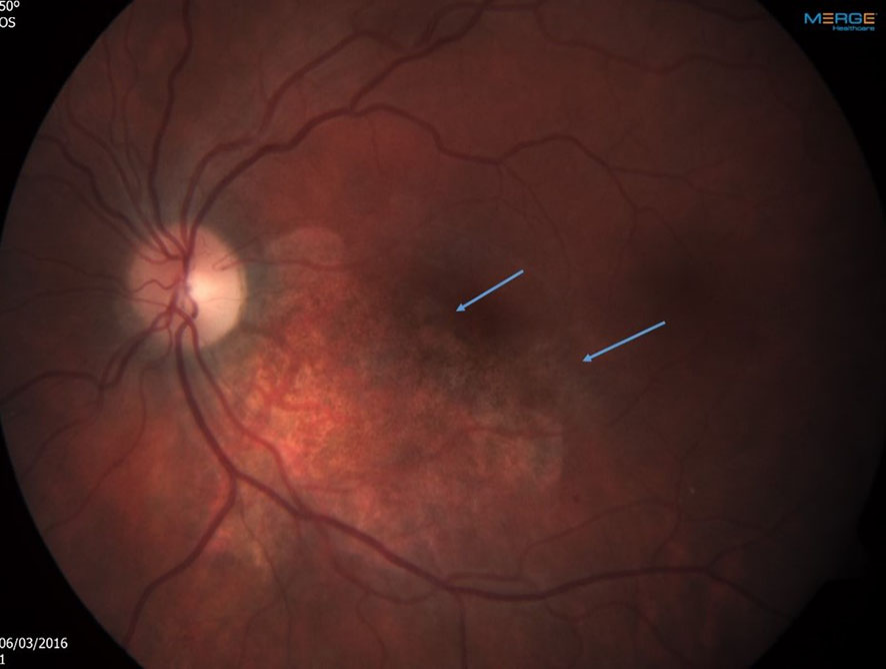
Left eye After 2 months of ambulatory intravitreal treatment. Note progression of the retinitis, threatening the central macula. The arrows point towards the perifoveal areas of active retinitis.

After 2 months of ambulatory intravitreal treatment, the retinitis showed further progression, threatening the central macula (**Figure 2**), and treatment was augmented with systemic foscarnet and immunoglobulins.

Similar to the solution for the patient’s refractory EBV lymphoma, it was assumed that his CMV-specific immunity needed to be reconstituted. Following international peer consultation, systemic pharmacologic anti-viral treatment was stopped and a third-party CMV-specific CTL was administered. In order to reduce the risk of reactive inflammation in the form of immune recovery vitritis, the intravitreal foscarnet injections were continued, and the new treatment was timed to coincide with the presence of saddle signs of active infection. Treatment protocol included 3 weekly infusions of 1×10^6/kg cytomegalovirus pp65 CTL.

There was a 2/8 HLA antigen matching, between donor cell lines and the recipient, which was considered by the cell bank as sufficient.

## 4 Outcome

**Figure 3:**
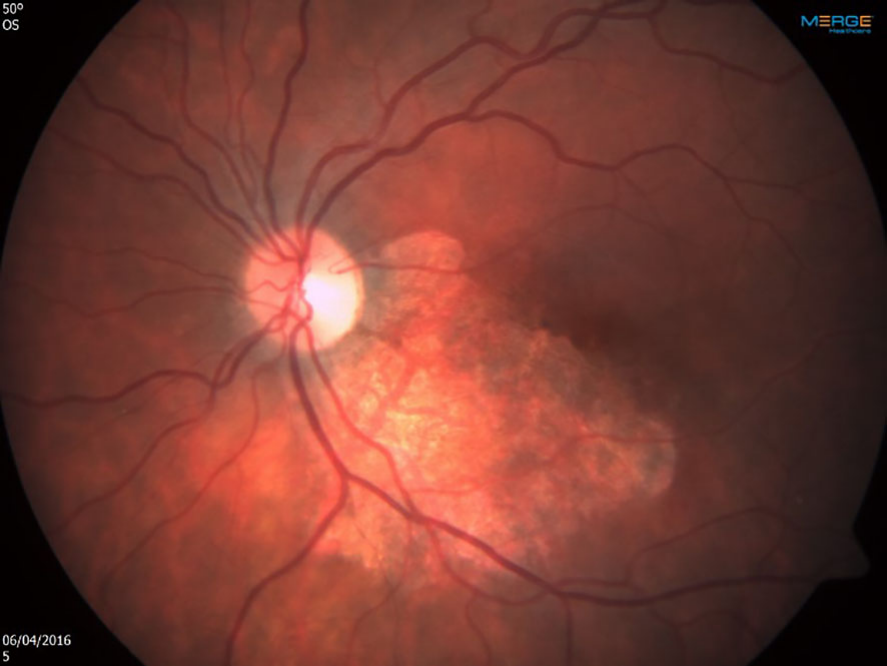
Left eye shortly after the first infusion of cytomegalovirus-specific cytotoxic T lymphocytes. Note the remarkable response, leading to complete resolution after second infusion.

Shortly after the first infusion of CMV-CTL, a remarkable response was noted. A few days after the second infusion, the retinitis resolved completely (**Figure 3**), there was no evidence of GVHD, nor was there an immune recovery uveitis. No recurrence was noted at the four-year follow-up, and since.

## 5 Discussion

This study report suggests that CMV-CTL may provide a long-term cure for both systemic and ocular CMV infection after allogeneic HCT, and that a prior CTL donation does not exclude a second donation of a different pathogen directed CTL.

CTL holds promise for the treatment of patients after allogeneic HCT who acquire drug-resistance emergence mutations consequent to anti-viral treatment. Since most hematopoietic cell donors are EBV- seropositive, the concept of providing virus-specific cellular immune therapy was initially applied by infusing un-manipulated donor lymphocytes. However, this strategy was found to be associated with an increased incidence of severe or fatal GVHD owing to the presence of alloreactive T cells in the infused cell product (5–7). Researchers suggested that the alloreactivity of the donor T cells could be reduced by selectively expanding the T-cell lines directed against specific viruses from the donor. A major disadvantage of this approach is that it requires the generation of virus-specific T lymphocytes in order to obtain the cell numbers needed for clinical use, and the manufacturing process takes approximately 3-4 weeks (8). Thus, it is not suitable for patients who need urgent therapy or who lack donor-derived virus-specific T cell options (recipients of cytomegalovirus-seronegative donor or cord blood grafts). A potential option in these cases is the use of third-party virus-specific CTL collected from virus-immune subjects with common HLA types, as biobanks of cryopreserved virus-specific CTL can be swiftly accessed. Published data suggest that despite the degree of mismatch inherent in this strategy, it is feasible, yields significant clinical responses, and does not precipitate high rates of graft-versus-host disease (GVHD) (9).

Another recent development is the generation of multiple-virus-specific T cells, each stimulated against (2–5) viruses (adenovirus, EBV, CMV, varicella zoster virus, and human herpesvirus) (6). If the line is appropriately matched with the recipient such that each virus is recognized through a shared HLA allele, this strategy offers treatment for a viral infection and prophylaxis against infection of several latent viruses in a single therapy (8, 10).

CMV-CTL and anti-viral drugs treats CMV reactivation/infection in two completely different methods. Anti-viral drugs exert their anti-CMV activity by inhibition of CMV UL54 DNA polymerase thereby disrupting CMV DNA synthesis. Viral mutations emerge in the setting of prolonged exposure to anti-viral drugs and lead to anti-viral resistance. The most common mutations are both mutations of the UL54 viral DNA polymerase and mutations of UL97 viral protein kinase, which phosphorylates ganciclovir and valganciclovir to an active form that inhibit UL54 viral DNA polymerase.

Infusion of CMV-CTL, on the other hand, restores patient’s immunity against CMV. After the infusion, the donor derived cells undergo expansion followed by improvement in proliferation ability and cytokine production. Donor derived cells also demonstrate long term persistence. In contrast, third-party donor CTLs are presumed to persist only transiently as expansion of these infused populations has been detected for 90 days. Yet, they still mediate durable responses. There are at least 3 possible explanations: they engraft at low levels beyond the threshold of detection and provide long-term protection; they simply provide a bridge until the endogenous virus-specific T cells recover; and/or they help to mediate recovery of the endogenous virus-specific T-cell responses (11, 12).

Former publications of cytotoxic T-lymphocyte infusion for the indication of CMV retinitis are sparse (13–16). The patient described in this report is unique because he was treated with a partially matched virus-specific T-cell donation from a third-party donor bank for two different viruses, at two time points. Each donation was supplied by a different donor, neither of whom was related to the patient or to the primary stem cell donor. The first donation was required to treat chemo-refractory EBV-induced B-cell lymphoma, and the second donation was used to treat drug-resistant CMV retinitis and viremia. In a recent publication describing the outcome of 190 patients treated for CMV infection with CMV-CTL donation (of whom 8 patients with CMV retinitis), patients who were previously treated with other adoptive cellular therapies were excluded 13. To the best of our knowledge, this is the first description of a single patient receiving CTL from two unrelated donors, directed for two different viruses for two separate clinical events.

Our patient had progressive CMV retinitis despite systemic and intravitreal drugs, and once there was immediate threats to the central vision area, we were forced to treat with a second CTL infusion, this time, directed against CMV, while continuing the anti-viral drugs. We were relieved to discover that immune recovery uveitis has not developed, and that the retinitis was cured with no recurrences in 4 years of follow up.

Studies verifying the safety and efficacy of adoptive immunotherapy with transplant-donor-derived, and a third party derived virus-specific CTL against latent viruses in patients after allogeneic HCT have prompted studies to extend this therapeutic alternative to a broader cohort of patients. The present case report adds to emerging evidence that retinitis can be safely and successfully managed with adoptive immunotherapy, even after a prior CTL donation was performed for a different indication.

## Data availability statement

The original contributions presented in the study are included in the article/**Supplementary Material**, further inquiries can be directed to the corresponding author.

## Ethics statement

Written informed consent was obtained from the individual(s) for the publication of any potentially identifiable images or data included in this article.

## Author contributions

All authors have no financial disclosures related to this study: TS, LS, ME-M, SP, ED, MY, MK. All authors contributed to the article and approved the submitted version.
