# Supplementary material for: Case Report: Cytomegalovirus-specific T-lymphocyte infusion for resistant cytomegalovirus retinitis
Source: Front Ophthalmol (Lausanne). 2023 Jul 3;3:1131674. doi: 10.3389/fopht.2023.1131674 (PMC11182297; doi:10.3389/fopht.2023.1131674)
Supplement: Supplementary file 1 [file DataSheet_1.docx]

12/17/2015

Sudden deterioration in the retinitis

Anterior chamber tap for PCR test.

The results confirmed the presence of a new-onset UL-97 mutation (viral resistance to ganciclovir and Valganciclovir)

Gancyclovir was replaced by twice weekly intravitreal injections of Foscarnet.

11/19/2015

Clinical regression of retinal lesions.

The intravitreal injections were tapered to once a week.

10/28/2015

Diagnosing CMV retinitis.

Started treatment with intravitreal Ganciclovir injections every 3 days (twice a week)

2/9/2016

The retinitis showed further progression, threatening the central macula and treatment was augmented with systemic foscarnet and immunoglobulins.

3/3/2016

Third-party CMV-specific CTL was administered. Intravitreal Foscarnet injections were continued.

The patient underwent 3 weekly infusions of 1×10^6/kg cytomegalovirus pp65 CTL.

3/12/2016

A few days after the second infusion, the retinitis resolved completely.
